# Molecular adaptations underlying high-frequency hearing in the brain of CF bats species

**DOI:** 10.1186/s12864-024-10212-6

**Published:** 2024-03-16

**Authors:** Xintong Li, Hui Wang, Xue Wang, Mingyue Bao, Ruyi Sun, Wentao Dai, Keping Sun, Jiang Feng

**Affiliations:** 1 College of Life Science, Jilin Agricultural University, Changchun, 130118 China; 2 Jilin Provincial Key Laboratory of Animal Resource Conservation and Utilization, Northeast Normal University, Changchun, 130117 China

**Keywords:** Bats, Brain, Echolocation, RNA-Seq, Adaptive evolution

## Abstract

**Background:**

The majority of bat species have developed remarkable echolocation ability, especially for the laryngeally echolocating bats along with high-frequency hearing. Adaptive evolution has been widely detected for the cochleae in the laryngeally echolocating bats, however, limited understanding for the brain which is the central to echolocation signal processing in the auditory perception system, the laryngeally echolocating bats brain may also undergo adaptive changes.

**Result:**

In order to uncover the molecular adaptations related with high-frequency hearing in the brain of laryngeally echolocating bats, the genes expressed in the brain of *Rhinolophus ferrumequinum* (CF bat) and *Myotis pilosus* (FM bat) were both detected and also compared. A total of 346,891 genes were detected and the signal transduction mechanisms were annotated by the most abundant genes, followed by the transcription. In hence, there were 3,088 DEGs were found between the two bat brains, with 1,426 highly expressed in the brain of *R. ferrumequinum*, which were significantly enriched in the neuron and neurodevelopmental processes. Moreover, we found a key candidate hearing gene, *ADCY1*, playing an important role in the *R. ferrumequinum* brain and undergoing adaptive evolution in CF bats.

**Conclusions:**

Our study provides a new insight to the molecular bases of high-frequency hearing in two laryngeally echolocating bats brain and revealed different nervous system activities during auditory perception in the brain of CF bats.

**Supplementary Information:**

The online version contains supplementary material available at 10.1186/s12864-024-10212-6.

## Background

Echolocation is the ability of an animal to determine its surroundings and target position by emitting sound waves and receiving echoes [1]. The echolocation behavior mainly relies on the emission process of ultrasonic signals and the auditory perception process of high-frequency echo acoustic waves [2, 3]. The bats brain can accurately sense surrounding environmental information by analyzing the signal differences between emitted sound waves and echogenic sound waves, including the acquisition of information, such as the distance from obstacles, shape and size, and texture, and can even accurately determine prey distance and flight speed [4–6].

With the developments of molecular phylogeny and high throughput sequencing technology, it is helpful for researchers to gain insights to the molecular bases underlying high-frequency hearing of echolocating bats. Typical echolocating bats relying on laryngeal vibrations for vocalization could be divided into two types, FM bats and CF bats. Compared with FM bats, CF bats have a higher dominant frequency of echolocation calls [7], unique CF components [8], highly sensitive structures to the dominant frequency on the basilar membrane of the cochlea [9], and unique Doppler frequency shift compensation behavior [10]. With the development of sequencing technology, the study on the evolution of high-frequency auditory adaptation in echolocation bats has entered the level of omics. Papers demonstrated that the molecular adaptations can occur not only in the coding sequence but also in the regulation of gene expression, where the level of gene expression is closely related to the demand for the protein and its functional necessity [11, 12].

Previously, significantly different expressed genes, along with different physiological processes, and adaptive evolutionary sites of hearing related genes were detected in the cochlea or inner ear of bats with different echolocating types [13]. Nearly, the cochlear structure and molecular bases underlying high-frequency hearing are well studied, however, lacking knowledge about the molecular adaptations of the bats brain [14].

The brain plays an important role in the processing of echolocating signals, and its auditory cortex contains higher-level auditory neural pathways [15]. At the molecular level, it has been found that the *Otof* gene functioning crucial in acoustic signal transmission in the brain of echolocating bats, and participating in the release of neurotransmitters at the synapse between inner hair cells and the auditory nerve [16]. Especially for the CF bats, developed different auditory nucleus specializations, including enlarged nuclei and cellular differentiation in the cochlear nucleus (CN) [17], distinctly different in the inferior colliculus (IC) region from other types of echolocating bats [18], medial geniculate nucleus (MG) neurons that are sensitive to CF acoustic signals [19], and delay-tuned neurons located in the auditory cortex are specialized [20]. Therefore, we suppose that significant genes and related physiological processes involving with high-frequency hearing could be present in the bat brain, and those genes could be differentially expressed in the brain of CF bats compared with FM bats.

Herein, we performed comparative brain transcriptome for the CF bat (*Rhinolophus ferrumequinum*, Rhinolophidae, dominant frequency: 74.70 ± 0.13 kHz) and FM bat (*Myotis pilosus*, Vesperonidae, dominant frequency: 38.21 ± 1.18 kHz) to identify the differentially expressed genes and associated physiological processes. For further, the key hearing related genes were detected and the following adaptive evolution analyses were conducted. This study provides further support for a comprehensive understanding of the widely molecular adaptations in the bat brain, especially to detect key genes and related physiological processes involving with high-frequency hearing in different types of echolocating bats.

## Materials and methods

### Sampling, RNA extraction and sequencing

The two bat species were both caught from a wild bat colony during September 2021 on the outskirts of Beijing, China (115°59′ N, 39°43′ E). Generally, transcriptome sequencing requires at least three biological repeats to evaluate the reproducibility among individuals within the same species, especially for wild animals. In order to minimize the influence on the wild bat populations and also meet the requirements of comparative transcriptome and statistical analyses, three individuals were collected as three biological repeats for each bat species, that is a total of six bat individuals were used in this study. To avoid any influence of sex-related differences, only males were selected. The brain tissues from each individual were collected and immediately flash-frozen in liquid nitrogen followed by placement in a − 80 °C freezer until processing for total RNA isolation.

Total RNA was isolated using the Total RNA extraction reagent (Trizol) in accordance with the manufacturer’s protocol. The quantity and quality of total RNA were measured using a Qubit 2.0 Fluorometer and gel electrophoresis. RNA samples of the same volume and concentration were used during the step of converting mRNA into cDNA. Three paired-end cDNA libraries of each species were generated using the mRNA-Seq assay. In total, 6 cDNA libraries were prepared at an equimolar ratio for transcriptome sequencing on the Illumina HiSeq ^TM^ platform. All raw reads were deposited in the NCBI Short Read Archive (SRA) Database under SRA accession. The raw sequence data generated were deposited into the NCBI Sequence Read Archive database (SRA run accession numbers: *R. ferrumequinum*: PRJNA1039161, *M. pilosus*: PRJNA1039163).

### Reference transcriptome assembly and functional annotation

The raw reads were filtered by Trimmomatic software [21] using six criteria: removing reads with adaptors; removing reads with unknown “N” bases; removing low-quality bases (Q value < 20) from Reads 3’ to 5’; removing low-quality bases (Q value < 20) from Reads 5’ to 3’; removing bases with quality values below 20 in the tail of Reads (window size of 5 bp) using the sliding window method; removing Reads with length less than 35nt and their paired Reads. To construct a common and powerful reference transcriptome for the comparative analyses, all high-quality raw reads from 6 individual cDNA libraries were used for de novo assembly by Trinity software [22, 23] with the default parameters. After the transcripts were reduced for sequence redundancy, the longest transcript in each cluster was taken as a unigene for further analysis. All of the remaining contigs are described as unigenes in the following text. The assembly result was evaluated by parameters such as the longest value of the sequence, the shortest value of the sequence, the N50 and the N90 values. To obtain the complete information on unigenes functional annotation, NCBI Blast + software [24] and KAAS [25] were used to compare the annotation results with 7 large databases, including NT, NR, COG, PFAM, CDD, GO and KEGG.

### Identification of differentially expressed genes (DEGs)

Considering the differences in library size, we first performed inter-sample normalization and used Bowtie2 software [26] to map clean reads to the assembled reference sequence. The expression levels of genes were calculated using the number of reads that were uniquely aligned with the reference transcriptome. Unique mapped reads were quantified into counts for each unigene and the transcripts per million (TPM) [27] formula method was used to determine the unigene expression level. The raw counts for each gene were converted into TPM value using the Salmon software [27] based on the following formula:

$$ {TPM}_{i}=\frac{{X}_{i}}{{L}_{i}}\times \frac{1}{{{\sum }_{j}\frac{{X}_{j}}{L}}_{j}}\times {10}^{6}$$

TPM was introduced in an attempt to facilitate comparisons across samples [28]. TPM stands for transcript per million, and the sum of all TPM values is the same in all samples, such that a TPM value represents a relative expression level that in principle should be comparable between samples [29]. Here, *Xi* denotes reads mapped to a transcript; *Li* is the transcript length, and the ${{\sum }_{j}\frac{{X}_{j}}{L}}_{j}$ corresponds to the sum of mapped reads to the transcript normalized by the transcript length.

The correlation coefficient between each pair of replicates for each sample was calculated using the R package (corrplot, version 0.92) to evaluate the reliability of the experimental results as well as the operational stability. To determine the separation of expression patterns across samples, principal component analysis (PCA)was performed on the levels of all unigenes using R package (vegan, version 2.6.4) to ensure more reliable results for subsequent analysis.

In consideration of less accurate genes with low expression levels, only unigenes with TPM ≥ 1 were retained for the following analyses. The DEGs between the *R. ferrumequinum* and *M. pilosus* were evaluated by DESeq2 (V1.12.4) software [30, 31]. In consideration of the general divergence among bat species and that the detection of DEGs would be more accurate for those with a greater difference in expression, a threshold of fold change ≥ 2 was in this study. Hence, to create a list of high-confidence DEGs for further analyses, the following stringent criteria were used: fold change ≥ 2, namely |log_2_ (fold change)| ≥ 1, in detail, the *P*-value was adjusted using the Benjamini-Hochberg method based on an FDR (False Discovery Rate) cut-off of 0.001 [32]. Visualization of the DEGs in the two bat species was achieved by creating a Volcano plot with the R package (ggplot2, version 2.2.1).

### GO category and KEGG pathway enrichment analyses

Downstream functional classification was achieved through the integrated localization of GO [33] and KEGG pathway databases [34]. We conducted the GO and KEGG enrichment analysis on the DEGs in OmicShare (http://www.omicshare.com/). All *P-values* were computed using the hypergeometric test, and multiple test correction was performed using the Benjamini–Hochberg method [35] based on *P-value* cut-off of 0.01.

To clarify the interactions between genes and key pathways, a directed acyclic graph [36] was subsequently constructed. Following GO and KEGG analysis of DEGs, the directed acyclic graph was plotted using the R package (topGO, version 2.50.0) [37] to visualize the GO terms generated by GO enrichment. GO enrichment is divided into three categories: cellular component, molecular function, and biological process [33]. For the results of KEGG enrichment analysis, function-function, and function-gene interaction network graphs were constructed by association analysis to identify key genes and key functions.

### Species coverage

Based on the above analyses, a candidate hearing related gene, *ADCY1* was identified as the core connected gene with highly connectivity with the pathways which were significantly enriched by highly expressed genes in the brain of *R. ferrumequinum*, implying that the *ADCY1* may play important roles in the brain of bats. Therefore, we performed following molecular evolution analyses to detect the adaptive evolutionary levels of the *ADCY1* gene in CF bats and also other echolocating bats and whales. In detail, the *ADCY1* gene sequence (698 bp) was obtained by RNA-Seq sequencing, after blasting in the NCBI database, the complete coding sequences (3,152 bp) of the *ADCY1* gene from 27 representative mammals were obtained. The species comprised of two CF bats (*R. ferrumequinum* and *Rhinolophus sinicus*), seven FM bats (*Molossus molossus*, *Pipistrellus kuhlii*, *Myotis myotis*, *Desmodus rotundus*, *Sturnira hondurensis*, *Phyllostomus hastatus*, *Phyllostomus discolor*), two nonecholocating bats (*Pteropus giganteus*, *Pteropus Alecto*), seven echolocating toothed whales (*Neophocaena asiaeorientalis asiaeorientalis*, *Phocoena sinus*, *Physeter catodon*, *Tursiops truncates*, *Orcinus orca*, *Lipotes vexillifer*, *Delphinapterus leucas*), two nonecholocating baleen whales (*Balaenoptera acutorostrata scammony*, *Balaenoptera musculus*) and seven other nonecholocating mammals (e.g., *Homo sapiens* and *Mus musculus*) (Supplementary Table S1).

### Phylogenetic reconstruction

Nucleotide sequences of the 27 species were aligned and spliced in the software ClustalX [38] and Bioedit [39], respectively. Tree construction used Neighbor-Joining (NJ) [40] as implemented in MEGA 11 [41], maximum likelihood (ML) using IQ-TREE [42], and Bayesian inference (BI) in MrBayes 3.2.2 [43]. For both ML and BI trees, the GTR + G nucleotide substitution model was used as the best model which was selected by the jModeltest 2.1.7 software [44], with bootstrap values of 100,000 replicates. For the BI trees, Markov Chain Monte Carlo (MCMC) data simulation was used to estimate the posterior probabilities and performed for 20 million generations with a sampling frequency of 1,000, including a burn-in step corresponding to the first 25% million generations.

### Molecular evolutionary analyses

To explore the heterogeneous selection pressures acting on each species, sliding window analyses were performed for *ADCY1* using SWAAP 1.0.2 [45]. We estimated the nonsynonymous substitution (dN) and synonymous substitution (dS) substitution rates (the dN/dS ratio, termed omega ω) according to the Nei and Gojobori method [46]. Window size and step size were set as 30 and 6 codons to determine the variations of selection pressure [47] along the *ADCY1* gene sequence for two classes of bat species, namely CF bats and FM bats.

The positive selection analysis needs to be performed based on a true species tree, so we determined the true genealogical occurrence relationships between the species used for this gene and mapped the species tree based on previous studies [3, 48, 49]. By comparing ω among sites and branches, the form and intensity of natural selection can be revealed, with ω < 1, ω = 1, and ω > 1 indicating negative selection, neutral evolution, and positive selection, respectively. Various models were analyzed using the CODEML program [50] of the PAML [51] package, including the Site Model (SM), the Branch Model (BM), and the Branch-Site Model (BSM). In the BM and BSM, five foreground branches were set up: CF bats branch, FM bats branch, non-echolocated bats branch, echolocated toothed whale suborder branch, and non-echolocated baleen whale suborder branch, denoted as Branch a, Branch b, Branch c, Branch d, and Branch e, respectively. In the positive selection analysis, the above five branches were analyzed as foreground branches, respectively, while the corresponding remaining branches were used as background branches.

Each Model includes alternative and null hypotheses, and Likelihood ratio tests (LRTs) are performed on the alternative and null hypotheses of the specific model based on the results of the corresponding model runs. The *P-value* of the LRTs result is 0.05, and if *P-value* < 0.05, the null hypothesis is rejected and the alternative hypothesis is accepted. LRTs is the test of significance, which means that the sites are considered positively selected sites when *P-value* < 0.05 and the Bayesian posterior probability is greater than 0.9.

### Localization of important sites

Combining the results of previous studies, we predicted and mapped the protein structure of the *ADCY1* gene schematically [52, 53]. The expression pattern of ADCY isoforms is mainly obtained from RNA sequencing analysis (at the mRNA level) [52], the *ADCY1* gene in this chapter study is a transmembrane protein gene. The protein domains and transmembrane topology of *ADCY1* were predicted and plotted according to InterProScan (https://www.ebi.ac.uk/interpro/) and TMHMM Server v. 2.0 (http://www.cbs.dtu.dk/services/TMHMM/). Subsequently, we mapped all the positively selected sites onto the schematic plot of the *ADCY1* protein to illustrate its potential changes in CF bats.

## Result

### Sequencing, assembly, and functional annotation

As shown by Table 1, there were 134,347,358 and 134,347,358 clean reads were obtained for *R. ferrumequinum* and *M. pilosus*, respectively (Table 1). The proportions of clean reads among raw reads in each library were 96.46% and 96.51%, respectively suggesting the high quality of the RNA-Seq data available for further analyses. A total of 346,891 unigenes sequences were obtained for the assembled reference transcriptome, ranging from 201 to 15,577 bp in length, with an N50 of 1,460 bp, N90 of 254 bp (Table 1), and the length of all unigenes were calculated (Supplementary Figure S1). After annotation, there were 17,760, 20,034, 14,299, 22,400, 37,912, 87,465, and 196,500 were respectively annotated by the CDD, PFAM, KEGG, KOG, GO, NR, NT databases, and 3,904 unigenes were successfully annotated in all databases (Supplementary Figure S2). For KOG annotation in particular, the term of signal transduction mechanisms was the most highly represented, followed by the transcription. (Supplementary Figure S3).

**Table 1:** Summary information of the brain transcriptome sequencing assemblies in *R. ferrumequinum* and *M. pilosus*

	*R. ferrumequinum*	*M. pilosus*
**Sequencing**		
Total Sequences (bp)	20,892,590,400	21,873,346,500
Total Reads Count (raw reads)	139,283,936	145,822,310
Total Reads Count (clean reads)	134,347,358	140,726,522
Ratio of clean/raw	96.46%	96.51%
**Assembly**		
Unigenes	346,891	
>=1000 bp	46,572	
N50	1,054	
N90	254	
Max Len	15,577	
Min Len	201	
Total Len	224,511,654	
Average Len	647	
**Mapping**		
Total mapped (%)	94.22%	95.48%
Unique mapped (bp)	27,976,748	37,451,281

**Brain molecular differences between** ***R. ferrumequinum*** **and** ***M. pilosus***.

**Fig. 1:**
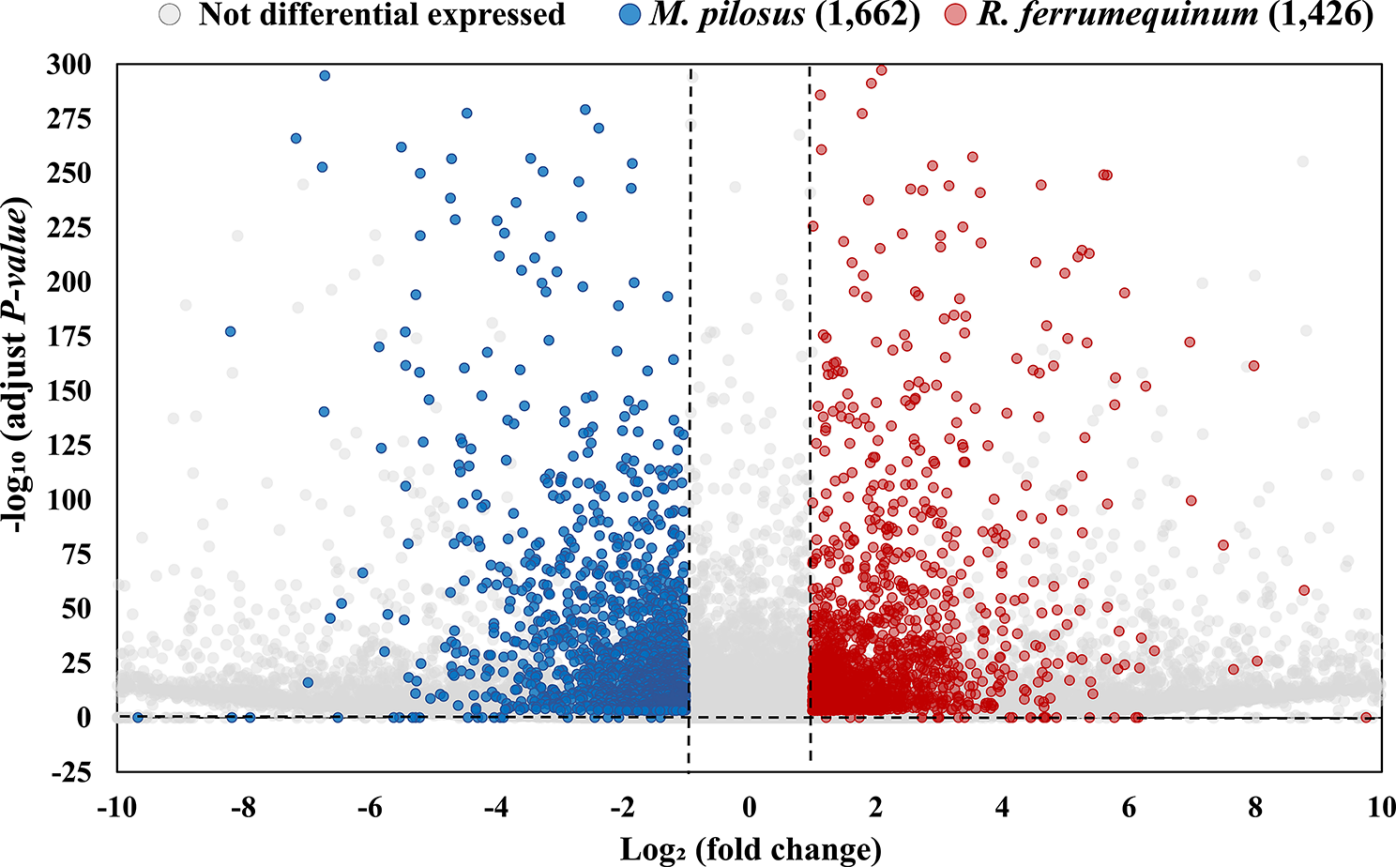
DEGs identified in the brains between the *R. ferrumequinum* and *M. pilosus* with|log_2_ (fold change)| ≥ 1 and adjust *P-value* ≤ 0.001

Results of PCA analysis, TPM density distribution and the correlation analysis of six bats brain samples (Supplementary Figure S4), consistently suggest that the samples show well repeatability and could be used for the downstream analyses. Accordingly, there were 3,088 DEGs were identified in the brains between the two bat species, including 1,426 highly expressed genes in *R. ferrumequinum* and 1,662 highly expressed genes in *M. pilosus* (Fig. 1).

**Fig. 2:**
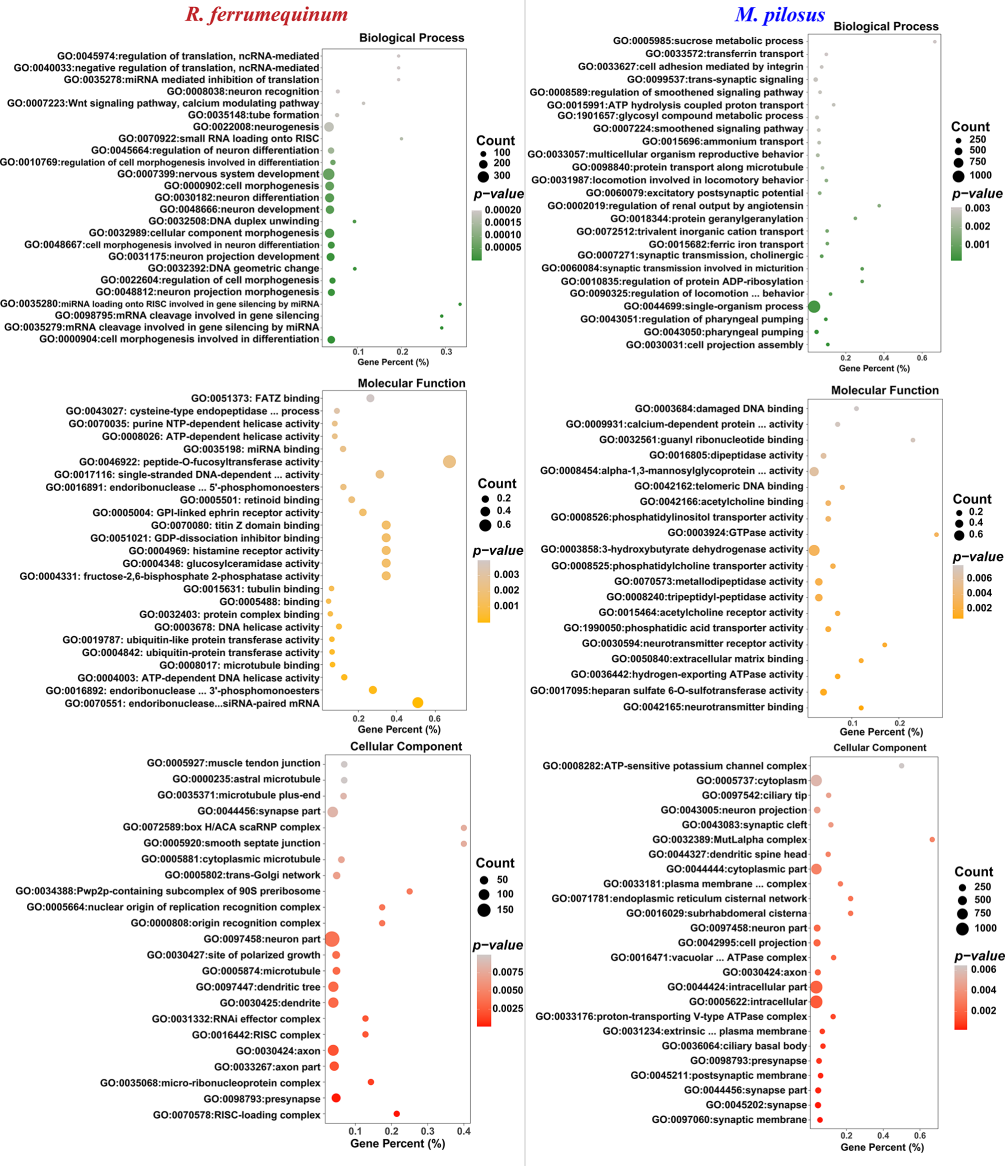
The GO terms significantly enriched by the up-regulated genes detected in *R. ferrumequinum* (left) and *M. pilosus* (right), respectively

**Fig. 3:**
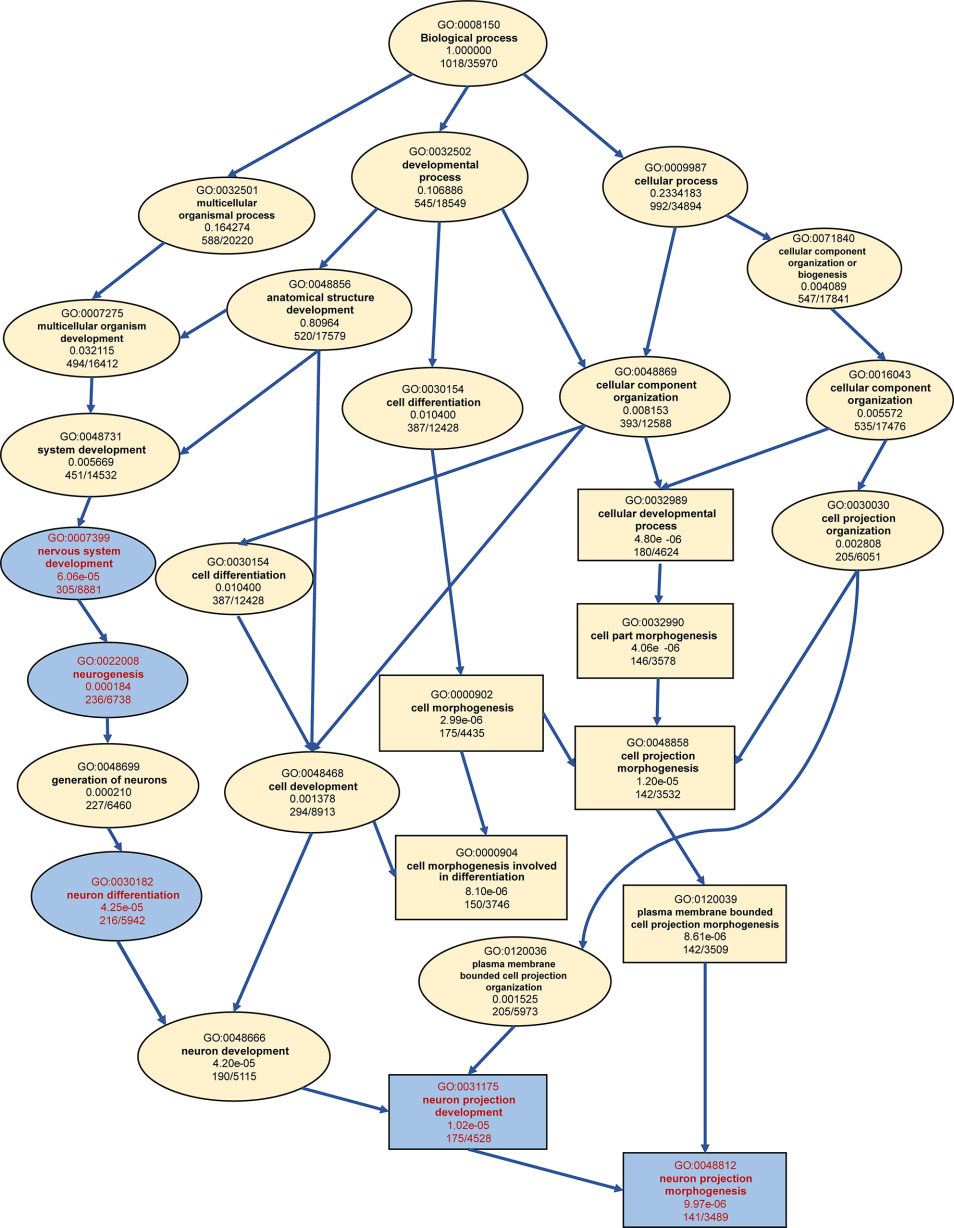
The directed acyclic graph for biological process GO terms significantly enriched by up-regulated genes detected in the brain of *R. ferrumequinum*. The circles represent GO terms, squares represent the top ten significantly enriched GO terms and arrows represent causal relationships. GO terms and GO terms associated with neural development in the same branch are marked in blue

**Fig. 4:**
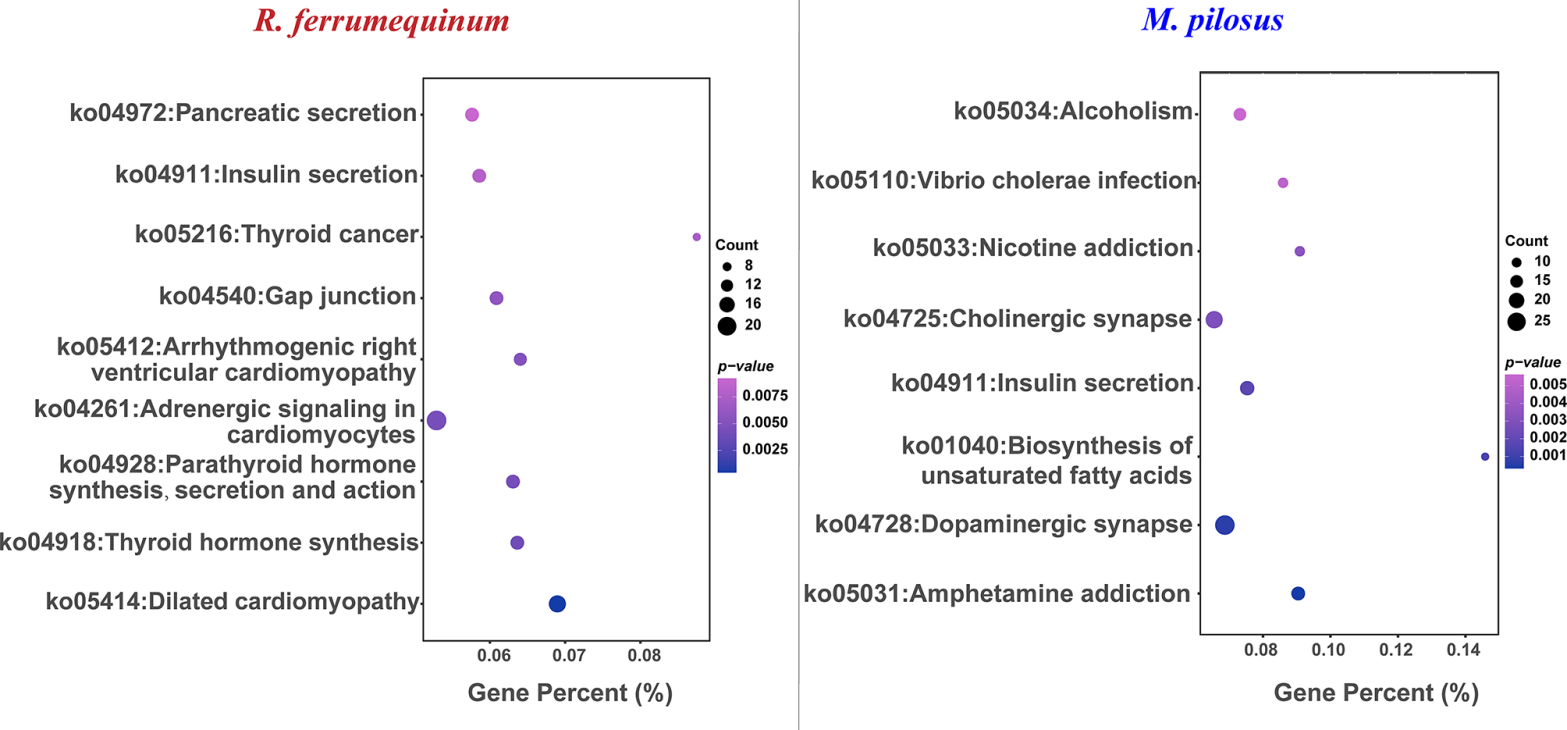
The KEGG pathways significantly enriched by the up-regulated genes detected in the brain of *R. ferrumequinum* (left) and *M. pilosus* (right), respectively

**Fig. 5:**
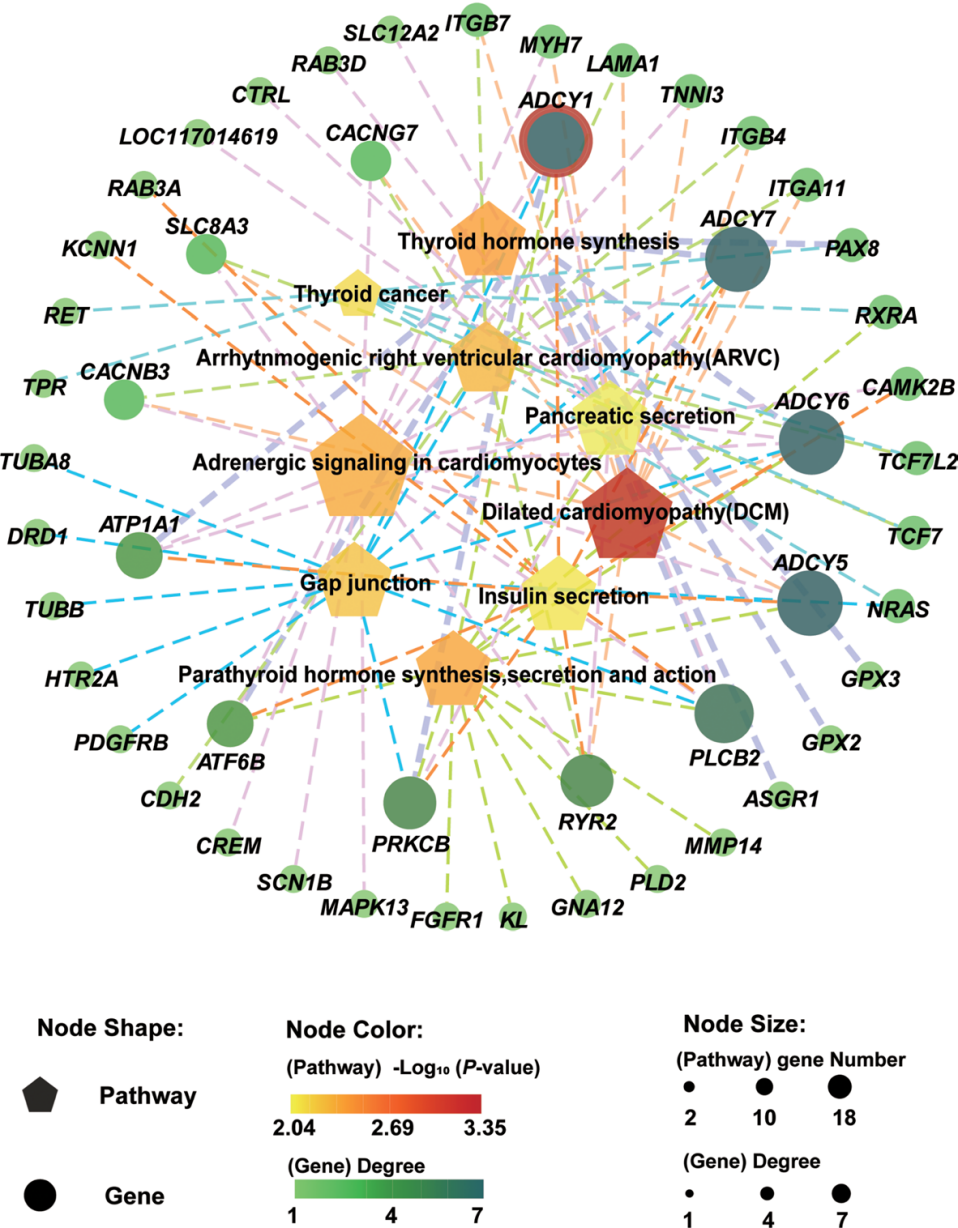
The KEGG pathway-gene interaction network analysis for *R. ferrumequinum*

DEGs identified between *R. ferrumequinum* and *M. pilosus* were significantly enriched in multiple GO terms and KEGG pathways (Supplementary Figure S5). The 1,426 highly expressed genes in the brain of *R. ferrumequinum* were significantly enriched in the GO terms of biological processes level, a large number of genes were enriched involving neurodevelopmental or cellular morphogenetic processes, such as nervous system development (GO: 0007399), neurogenesis (GO: 0022008), cellular component morphogenesis (GO:0032989), neuron differentiation (GO:0030182), etc. Refer to the 1,662 highly expressed genes in the brain of *M. pilosus*, they were significantly enriched in the GO terms of regulation of pharyngeal pumping (GO: 0043051) and cell projection assembly (GO: 0030031), pharyngeal pumping (GO:0043050), single-organism process (GO:0044699) etc., which were different with the corresponding results of *R. ferrumequinum* (Fig. 2). Furthermore, the directed acyclic graph showed that the nervous system development (GO: 0007399), neurogenesis (GO: 0022008), neuron differentiation (GO: 0030182), neuron projection development (GO: 0031175), neuron projection morphogenesis (GO: 0048812) terms were in the same branch (Fig. 3). In the molecular function level, most of the terms which were significantly enriched by the highly expressed genes detected in *R. ferrumequinum* were related with enzymatic activity, suggesting that more chemical reactions requiring enzymatic catalysis may exist in the brain of *R. ferrumequinum.* Whereas, for *M. pilous*, terms were mostly related with channel activity and receptor activity, etc. (Fig. 2). Whereas, different situations were conducted for the GO terms of cellular component, highly expressed genes detected in *R. ferrumequinum* and *M. pilosus* were also significantly enriched in similar terms, including neuron part (GO:0097458), synapse part (GO:0044456), axon (GO:0030424), presynapse (GO:0098793) (Fig. 2). The results of the KEGG enrichment analysis showed that *R. ferrumequinum* was significantly enriched in pathways related to Thyroid hormone synthesis, etc. In addition, both CF and FM bats were enriched to Insulin secretion (ko04911) (Fig. 4). The network interaction analysis conducted by the significantly enriched KEGG pathways showed that Thyroid hormone synthesis (ko04918) was the key pathway in *R. ferrumequinum*. Moreover, we found the *ADCY1* gene, one candidate hearing related gene, was the most highly connected gene in the brain of *R. ferrumequinum* revealed by the functional-gene network interaction diagram (Fig. 5).

### Adaptive evolution of the *ADCY1* gene

**Fig. 6:**
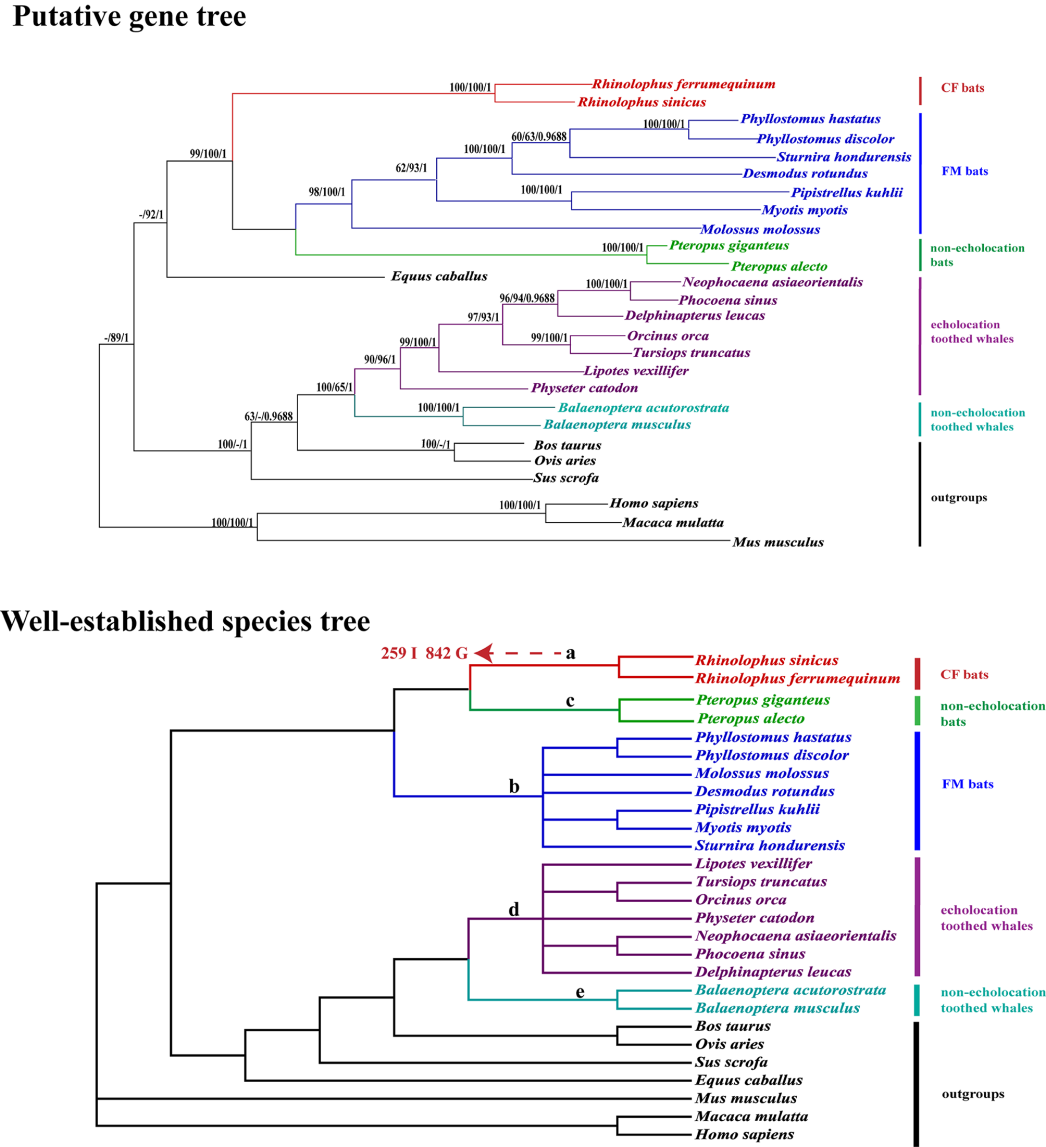
Putative gene tree and well-established species tree for the 27 mammal species, respectively

In total, coding region sequences of the *ADCY1* gene for 27 mammals were successfully collected (Supplementary Table S1). Putative gene tree of the *ADCY1* gene based on NJ, ML and BI methods showed similar topological structures and high support was obtained at most of the species branch nodes (Fig. 6). In addition, all methods support that CF bats can be clustered together separately, and then clustered together with FM bats and Click bats, which produced a tree topology slightly different from previously reported mammalian species trees (Fig. 6). The Putative gene tree constructed from the *ADCY1* gene set consisting of 27 species clustered CF bats together individually, while FM bats and non-echolocated bats clustered at the periphery, and the above clusters had high statistical support. The true species relationship, however, is that CF bats cluster together with non-echolocating bats before clustering with FM bats, demonstrating that the gene evolved differently in CF bats than in other species.

**Putative gene tree** for *ADCY1* coding region sequences using Neighbor-Joining (NJ), maximum likelihood (ML) and Bayesian inference (BI). Values on the branch indicate support from NJ, ML and BI, respectively.

**Well-established species tree** [3, 48, 49] based on the basis of previous studies for the 27 species. Red amino acid substitutions with ω > 1 are showed. Letters from a–e indicate branches to be tested under Branch model or Branch-site model.

### Molecular evolution analyses for the *ADCY1* gene

**Fig. 7:**
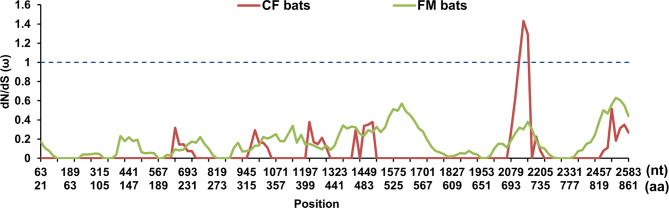
The ω-value distribution map of *ADCY1* gene in FM bats and CF bats based on sliding window analysis

Sliding window analysis showed that the ω values were greater than 1 in the CF bats clade only, demonstrating that the gene suffered stronger selective pressures than the FM bats (Fig. 7). As the result of PAML analyses, Site Model (SM) found six positively selected sites for *ADCY1* gene but only one site (242 K 0.905) were significant (supplementary Table S2). The result of the Branch Model (BM) showed that only the ω values of the CF bats branch (ω1) were greater than those of the other remaining branches, implying different evolutionary rates among the CF bats and other mammals (supplementary Table S3). Two positively selected sites (259 I 0.837 and 842 G 0.518) were found by Branch-Site Model (BSM) when CF bats setted as the foreground branch however the *P-value* of LRT test was not significant (Supplementary Table S4).

**Fig. 8:**
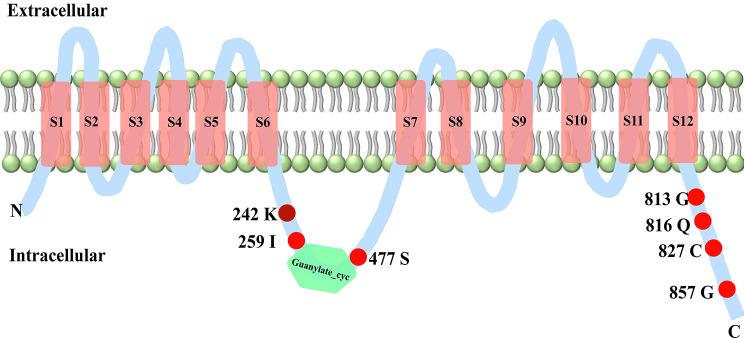
Schematic plot for *ADCY1* protein structure

The *ADCY1* protein consists of 12 transmembrane segments (S1 – S12), the structural domain Guanylate_cyc contains 161 amino acid sites between the transmembrane regions S6 and S7. Then, we mapped the potential significant positively selected sites onto the protein structure and found that the significant positive selection sites 242 K, 259 I, and 477 S were located close to Guanylate_cyc, while the other positive selection sites were all located at the C-terminus of the intramembrane region of *ADCY1* protein (Fig. 8).

Red rectangles cover the twelve transmembrane segments (S1 – S12) and the green hexagon represent one structural domain (Guanylate_cyc) between the S6 and S7. Red points with labeled numbers indicate nominally positively selected sites detected by PAML, and the site of 242 K is a significant positively selected site.

## Discussion

As was first proposed several decades ago, alterations (or innovations) in gene expression are regarded as an essential means of generating biological diversity [11, 54]. Therefore, analyses of DEGs can reveal the molecular mechanisms underlying phenotypic diversity and provide a deeper understanding of the relationship between gene expression patterns and the resultant phenotypes [55, 56]. We have performed the comparative brain transcriptome between *R. ferrumequinum* and *M. pilosus*, using Illumina sequencing technology. A total of 346,891 reference transcriptomes were obtained. In addition, differences in expressed genes and associated physiological processes were found in the two bat brains with different types of echolocating types and two important insights based on downstream analyses were summarized.

### Different brain neural activities between CF and FM bats

The results of this study suggest that there may be different neural activity in brain in *R. ferrumequinum* (CF bat) compared with *M. pilosus* (FM bat). On the one hand, the results of GO enrichment analysis biological process categories showed that highly expressed genes in brain of *R. ferrumequinum* were significantly enriched in GO terms directly related with nervous system development. Especially, some of the GO terms enriched by the highly expressed genes in the brain of *R. ferrumequinum*, such as nervous system development (GO: 0007399), neurogenesis (GO: 0022008), neuron differentiation (GO: 0030182), neuron development (GO: 0031175), neuron projection morphogenesis (GO: 0048812) terms were in the same branch revealed by the directed acyclic graph. On the other hand, Genes highly expressed in the *R. ferrumequinum* brain were significantly enriched in pathways related to thyroid hormone synthesis (ko 04918) which play a critical role in the differentiation, development of the central nervous system as well as the formation of various functions [57], indicating that the brains of the CF bats may present more active neural activity rather than the FM bats.

Echolocation principal frequency, as an important acoustic parameter, carries a lot of important individual information, and there are large differences in principal frequencies between different types of echolocation bat species [58–60]. Bat species with different echolocation types and frequencies have evolved over a long period to be able to perceive well the echolocation acoustic frequencies emitted by themselves [61, 62]. The dominant frequency of the CF bats is significantly higher than the FM bats. The dominant frequencies of CF bat (*R. ferrumequinum*) and FM bat (*M. pilosus*) in this study are 74.70 ± 0.13 kHz and 38.21 ± 1.18 kHz, respectively. The perception of echolocation acoustic signals begins in the cochlear hair cells, is continuously distributed on the auditory nerve and terminates in the auditory cortex of the brain [63]. Therefore, we speculate that differences in echolocation principal frequencies is one of the reasons for the differences in gene expression in the brain of different types of echolocating bat species. In addition, there are neural specializations within the auditory brainstem and cortex of echolocating bats [18], such as delay-tuned neurons [64, 65]. In particular, the tonotopic organization of the auditory regions [19], the dorsal and abdominal hypothalamic regions of CF bats are different from other types of echolocating bats and other mammals [66]. The differences described above in CF bats brain are likely the result of adaptive changes to adapt to their unique high-frequency CF component in the echolocation calls. Compared with FM bats, genes highly expressed in the CF bats brain mostly involving with nervous system development related biological processes, which is likely to be the genetic basis of those specific structures and functions in CF bats brain. It has been shown that the auditory cortex of CF bats has some well-developed physiology and organization to respond to specific neurons compared to the auditory cortex of other mammals [19], since then, we suppose that there may be specific types of neurons in CF bats’ brain, and the above highly expressed genes and associated biological functions may be a direct reflection at the molecular level.

Besides, although the highly expressed genes were different in the CF bats compared with FM bats, the same pathway, Insulin secretion (ko04911), was also detected to be significantly enriched by DEGs detected in CF and FM bats, respectively, indicating there were similar biological processes and molecular mechanisms in the brains for recognizing CF and FM bats. Insulin acts on the CNS to modulate behavior and systemic metabolism [67]. Recently, the *WFS1* gene expressed in the brain involving with insulin secretion has attracted extensive attention [68], and the heterozygous mutations in the *WFS1* gene may cause deafness [69], hence this gene and its associated pathways, like insulin secretion, may be a possible mechanism for different hearing development.

### Adaptive evolution of hearing-related gene *ADCY1*

Previous studies have demonstrated that the *ADCY1* gene is a member of the adenylate cyclase gene family and primarily expressed in the brain, in details, with high expression levels in neurons and moderate in oligodendrocytes, microglia and astrocytes [52, 70]. The *ADCY1* gene was also detected to express throughout inner ear development and maturation in mouse [71]. In this study, The *ADCY1* gene was identified to be the core connected gene with highly connectivity with the pathways which were significantly enriched by highly expressed genes in the brain of *R. ferrumequinum*, indicating its important functions in the auditory process in echolocating bat. Therefore, following adaptive evolutionary analyses of the *ADCY1* gene were performed to further reveal its potential changes of functional sites along the sequence in echolocating bats. A multi-model positive selection analysis of the *ADCY1* gene identified several positive sites with highly significant 242 K 0.905, and the remaining sites were detected with posterior probabilities not reaching 0.9, suggesting that these sites may be evolutionarily important sites for the *ADCY1* gene, but have not yet reached significant positive selection at this stage. Not many sites were detected in the positive selection analysis, indicating that the gene is relatively conserved during evolution in CF bats, but functional gene sequences with important roles are usually conserved to ensure the stability of gene function in genetic evolution [72–74].

Combined with the results of the sliding window analysis (Fig. 7), the *ADCY1* gene was subjected to inconsistent selection pressure in the two species, with ω>1 occurring only in the CF bats category, demonstrating that a locus in this part of the bat may have been subjected to positive selection in CF bats and that most of the positively selected sites were at locations where non-synonymous and synonymous substitutions of *ADCY1* amino acids occurred frequently. Previously, hearing-related genes, *sk2* and *shh* were also detected by the sliding window analyses hand with stronger selection pressure in echolocation bats, and their potential positive selection sites were found in the CF bats branch, but both genes were evolutionarily relatively conserved [75].

By the results of structural and molecular evolutionary analysis of *ADCY1* protein, we found that all positive selection sites were distributed in parts outside the functional domain. The protein structure-function prediction revealed that most of the sites subject to positive selection are located near the structural domain or at the C-terminus. The main reason may be that since the key functional domains of channel proteins are responsible for exercising important transport functions, the protein sequences of these important functional domains have evolved more conservatively across species to ensure proper ion transport and signaling, while the positive selection sites detected in the non-important functional domain fraction may be the sites that exert an influence on the evolution of high-frequency hearing in CF bats. The structural domains of genes may be more conserved in gene sequence to ensure the stability of gene function during inheritance due to their decisive role in the exercise and maintenance of the overall gene function [74].

All of these results suggest that the auditory-related gene *ADCY1* has undergone adaptive evolution in CF bats species. Considering that CF bats have higher vocalization frequencies and auditory abilities compared to other echolocation species, it is hypothesized that potential positive selection sites detected on the *ADCY1* gene may play an important role in the acquisition and development of their echolocation abilities or high-frequency auditory abilities. Although *ADCY1* is evolutionarily conserved at the sequence level, compared with *M. pilosus*, it is highly expressed in *R. ferrumequinum* brain. Therefore, we suspect that *ADCY1* may be act by increasing the expression level, rather than through the changes at the sequence level for now.

In addition, we found the auditory gene *Otof* [16] and the vocal gene *FOXP2* [76] were also highly expressed in CF bats, although not significantly so. The above results demonstrate the presence of key genes related to hearing in the brain of CF bats, which may be responsible for the ability of CF bats to receive higher-frequency sound waves. However, the well studied hearing genes, *Prestin* [12, 77], *KCNQ4* [72, 78] and *SK2* [75, 79], which were previously reported to have undergo adaptive evolution in the laryngeal echolocation bats cochlea, were not detected in these two bats brains. Together, we speculated different molecular mechanisms may exist in the brain and cochlea in sensing high-frequency sound waves, reflecting by expressed different hearing related genes, different expression modes, and different adaptive sites.

The auditory-related gene *ADCY1* in this study is the first to be identified in the comparative transcriptome of the bat brain, complementing previous findings in the auditory system. We have found the *ADCY1* gene in the brain, which enriches the previous adaptive evolution research carried out by auditory genes.

## Conclusions

Neuron, synapse parts and other related genes were detected to be widely expressed in the brains of two typical laryngeally echolocating bat species. In particular, compared with FM bats, much more highly expressed genes were found to significantly enriched in neurodevelopment and thyroid hormone synthesis suggesting more active nervous activity in the brain of CF bats. Similar results were also demonstrated by the study of the cochleae between CF bats and FM bats. Moreover, a key gene, *ADCY1* was identified to be the most highly connected gene, and downstream analyses indicated that the *ADCY1* gene has suffered more powerful selective pressures and may act important functions in the brain of CF bats. This study provides further support for a comprehensive understanding of the expressed genes in the brain involved in bats echolocation with a view to uncover the molecular bases of high-frequency hearing in echolocating bats. In future, further analyses and correspondingly functional verification experiments would be performed on other types of bats to explore the accurately molecular mechanisms of high-frequency hearing in laryngeally echolocating bats.

### Electronic supplementary material

Below is the link to the electronic supplementary material.

Supplementary Material 1

Supplementary Material 2

Supplementary Material 3

Supplementary Material 4

Supplementary Material 5

## Data Availability

Raw data are available at SRA, under the accession number: PRJNA1039161 and PRJNA1039163.

## Abbreviations

DEGs: differentially expressed genes
CN: cochlear nucleus
IC: inferior colliculus
MG: medial geniculate
LRTs: Likelihood ratio tests
