# Supplementary material for: Molecular adaptations underlying high-frequency hearing in the brain of CF bats species
Source: BMC Genomics. 2024 Mar 16;25:279. doi: 10.1186/s12864-024-10212-6 (PMC10943862; doi:10.1186/s12864-024-10212-6)
Supplement: Supplementary file 1 — Supplementary Material 1 [file 12864_2024_10212_MOESM1_ESM.pdf]

Supplementary Materials

Figure S1 Length distribution of distinct assembled Unigenes.

Figure S2 Venn diagram of CDD, PFAM, KEGG, KOG, GO, Nr, and Nt databases showing homology sequence numbers of the Unigenes in the seven different databases.

Figure S3 KOG functional classification of the Unigenes. The x-axis indicates the categories and the y-axis indicates the number of Unigenes.

Figure. S4 Reslts of sample TPM distribution, PCA, and correlation analysis of all brain samples used in this study.

A. TPM density distribution of Unigenes in the brain of *R. ferrumequinum* and *M. pilosus*.

The x-axis indicates the $\log_2$ (TPM) and the y-axis indicates the relative density value. Each color in the plot represents a sample, each region has an area of 1. The peak value of the density curve represents the region with the most concentrated Unigenes expression in the whole sample.

B. Principal component analysis (PCA) of the transcriptome in the brain of *R. ferrumequinum* and *M. pilosus*.

The numbers in parentheses represent the proportion of variance explained by that principal component. PC1, PC2, PC3 represent the top three dimensions of the genes showing differential expression among these samples, which account for 58.83%, 10.7% and 10.41% of the expressed genes, respectively.

C. Correlation analysis of each sample from *R. ferrumequinum* and *M. pilosus*.

The right and lower sides are the sample names, the left and upper sides are cluster situations.

Figure. S5 Statistics of the number of significantly GO terms and KEGG pathways significantly enriched by up-regulated genes detected in the brain of *R. ferrumequinum* and *M. pilosus*, respectively.

Table S1 Basic information of the 27 mammal species used for the adaptive evolutionary analysis.

Table S2 Result of Site model (SM) analysis for the *ADCY1* gene based on the species tree.

Table S3 Results of Branch model (BM) analysis for the *ADCY1* gene based on the species tree.

Table S4 Results of Branch site model (BSM) analysis for the *ADCY1* gene based on the species tree.

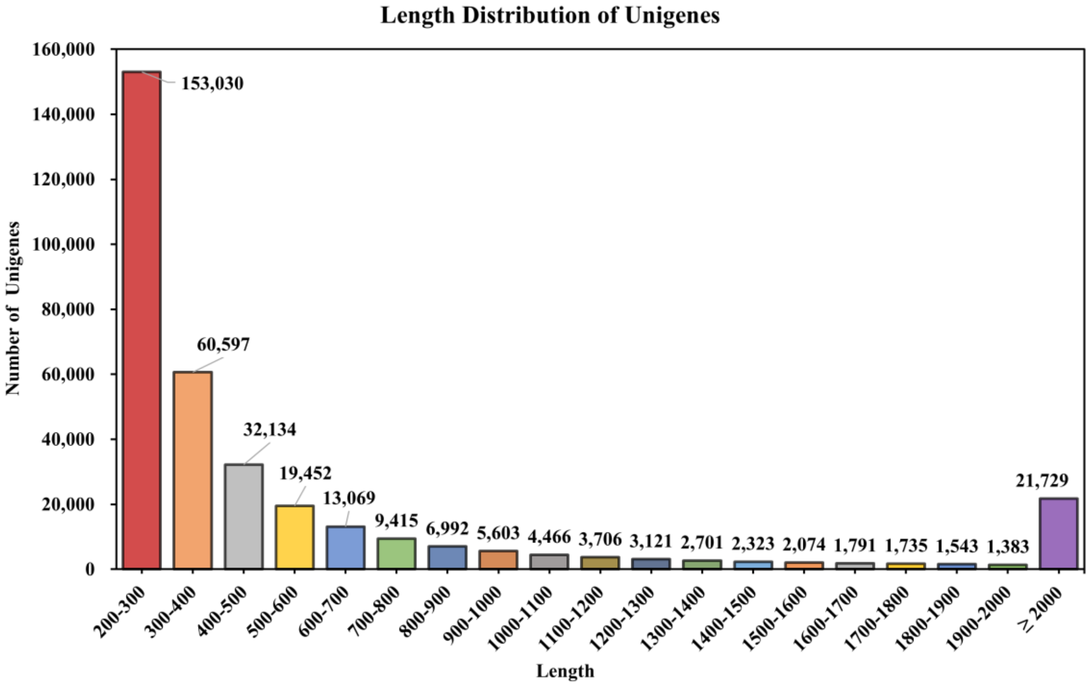

Figure S1 Length distribution of distinct assembled Unigenes.

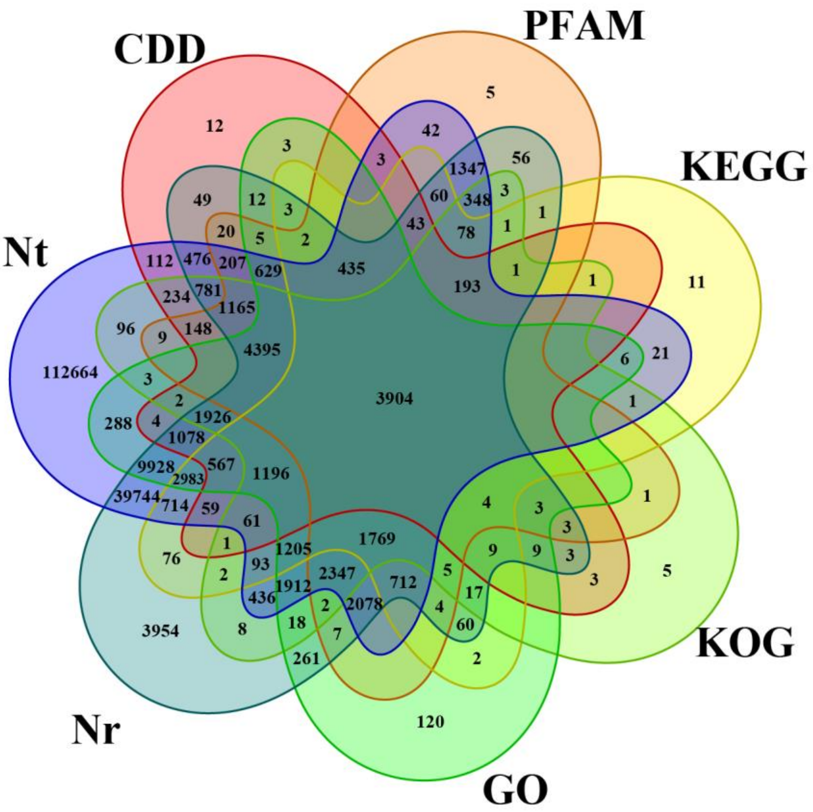

Figure S2. Venn diagram of CDD, PFAM, KEGG, KOG, GO, Nr, and Nt databases showing homology sequence numbers of the Unigenes in the seven different databases.

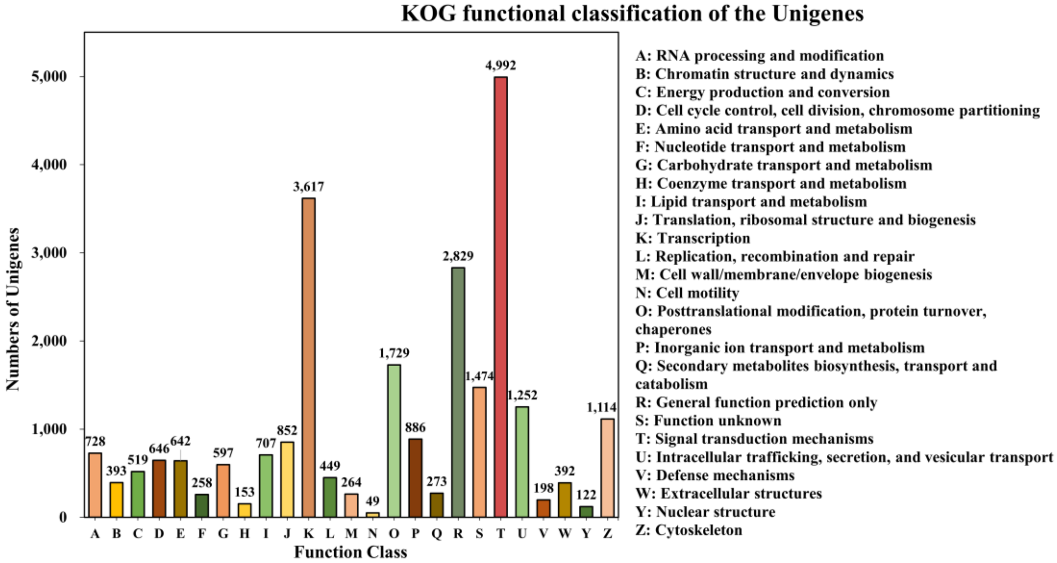

Figure S3. KOG functional classification of the Unigenes.

The x-axis indicates the categories and the y-axis indicates the number of Unigenes.

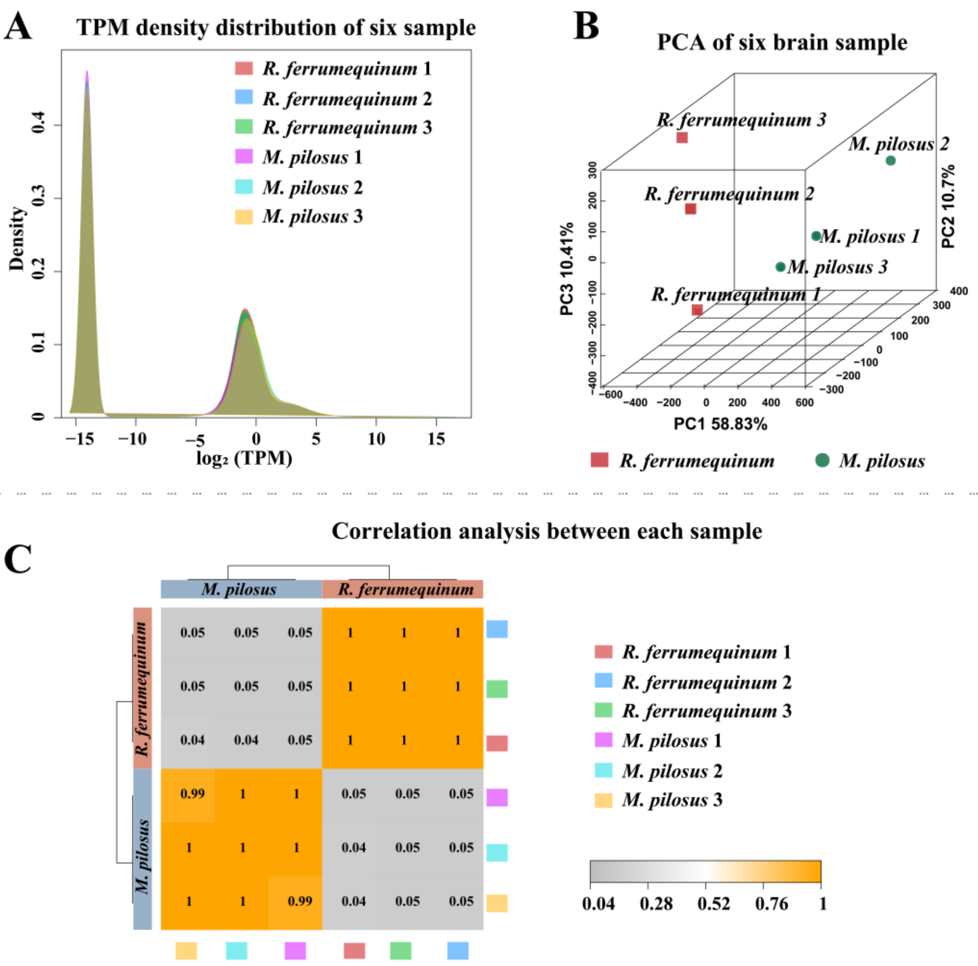

Figure S4 Reslts of sample TPM distribution, PCA, and correlation analysis of all brain samples used in this study.

A. TPM density distribution of Unigenes in the brain of *R. ferrumequinum* and *M. pilosus*.

The x-axis indicates the $\log_2$ (TPM) and the y-axis indicates the relative density value. Each color in the plot represents a sample, each region has an area of 1. The peak value of the density curve represents the region with the most concentrated Unigenes expression in the whole sample.

B. Principal component analysis (PCA) of the transcriptome in the brain of *R. ferrumequinum* and *M. pilosus*.

The numbers in parentheses represent the proportion of variance explained by that principal component. PC1, PC2, PC3 represent the top three dimensions of the genes showing differential expression among these samples, which account for 58.83%, 10.7% and 10.41% of the expressed genes, respectively.

C. Correlation analysis of each sample from *R. ferrumequinum* and *M. pilosus*.

The right and lower sides are the sample names, the left and upper sides are cluster situations.

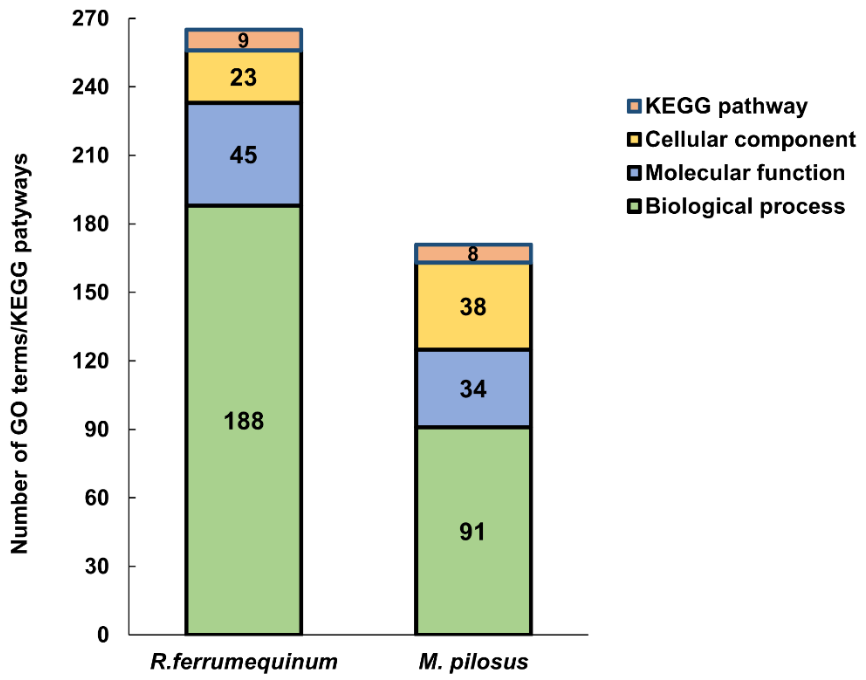

Figure S5 Statistics of the number of significantly GO terms and KEGG pathways significantly enriched by up-regulated genes detected in the brain of *R. ferrumequinum* and *M. pilosus*, respectively.

Table S1 Basic information of the 27 mammal species used for the adaptive evolutionary analysis.

**Order**	**Family**	**species**	**Sequence source**
Chiroptera	Rhinolophidae	*Rhinolophus ferrumequinum*	XM_033088417.1:17-2584
		Rhinolophus sinicus	XM_019731617.1:120-2018
	Molossidae	*Molossus molossus*	XM 036247217.1:17-3403
	Phyllostomidae	*Desmodus rotundus*	XM 024562866.2:358-3081
		Sturnira hondurensis	XM_037041048.1:16-3444
		Phyllostomus hastatus	XM_045827604.1:1-3417
		Phyllostomus discolor	XM_028504202.2:1-3435
	Vespertilionidae	*Pipistrellus kuhlii*	XM 036440145.2:35-3391
		Myotis myotis	XM 036327192.1:1-3393
	Pteropodidae	*Pteropus alecto*	XM_025046483.1:186-2885
		Pteropus giganteus	XM 039847110.1:1-3393
Artiodactyla	Delphinidae	*Tursiops truncatus*	XM_033862808.1:17-3421
		Orcinus orca	XM_012537851.3:101-3505
	Phocoenidae	*Neophocaena asiaeorientalis asiaeorientalis*	XM 024745779.1:401-3124
		Phocoena sinus	XM 032643229.1:101-3505
	Lipotidae	*Lipotes vexillifer*	XM_007447025.1:79-3483
	Monodontidae	*Delphinapterus leucas*	XM 022593147.1:781-3504
	Physeteridae	*Physeter catodon*	XM_007109833.2
	Balaenopteridae	*Balaenoptera musculus*	XM_036863914.1:101-3511
		Balaenoptera acutorostrata scammoni	XM_007187686.2:242-2965
Artiodactyla	Suidae	*Sus scrofa*	XM_021078661.1:1-3411
	Bovidae	*Bos taurus*	M25579.1:103-3507
		Ovis aries	XM_027968689.2:1-3405
Primates	Hominidae	*Homo sapiens*	NM_021116.4:177-3536
	Cercopithecidae	*Macaca mulatta*	XM_015133591.2:1-3357
Perissodactyla	Equidae	*Equus caballus*	XM_005614861.3:614-3337
Rodentia	Muridae	*Mus musculus*	NM_009622.2:168-3524

Table S2 Result of Site model (SM) analysis for the *ADCY1* gene based on the species tree.

**Site model (SM)**									
**Model**	**np**	**Ln L**	**Estimates of parameters**				**Model compared**	**LRT P-value**	**Positive sites**
M3	52	-14554.437871	p:	0.67833	0.25795	0.06372	M0 vs. M3	0.000000000	
			ω:	0.00157	0.12701	0.45268			
M0	48	-14927.624142	ω0:	0.05410					Not Allowed
M2a	51	-14754.734504	p:	0.93478	0.06522	0.00000	M1a vs. M2a	0.998359347	
			ω:	0.03673	1.00000	49.72458			
M1a	49	-14754.732862	p:	0.93478	0.06522				Not Allowed
			ω:	0.03673	1.00000				
M8	51	-14568.467978	p0=0.97966	p=0.12899	q=1.39481		M7 vs.M8	0.000007568	**242 K 0.905**, 477 S 0.615, 813 G 0.834,816 Q 0.665,827 C 0.758,857 G 0.875
			(p1= 0.02034)	ω= 1.00000					
M7	49	-14580.259606	p=	0.15263	q=	1.41633			Not Allowed
M8a	50	-14568.467978	p0=0.97966	p=0.12898	q=1.39468		M8a vs.M8	1.000000000	Not Allowed
			(p1= 0.02034)	ω= 1.00000					

Table S3 Results of Branch model (BM) analysis for the *ADCY1* gene based on the species tree.

Model	np	Ln L	Estimates of parameters			Model compared	LRT P-value
**Two ratio Model 2**	53	-14925.917530	ω:	ω0=0.05516	ω1=0.06657,		
					ω2=0.04631,		
					ω3=0.04984,	Model 0 vs. Two ratio Model 2	0.636556668
					ω4=0.02621,		
					ω5=0.03676		
Model 0	48	-14927.624142	ω=	0.05410			

Table S4 Result of Branch site model (BSM) analysis for the *ADCY1* gene based on the species tree.

**Branch site model (BSM)**									
**Model**	**np**	**Ln L**	**Estimates of parameters**					**LRT P-value**	**Positive sites**
**Branch a**									
Model A	51	-14752.399708	Site class	0	1	2a	2b		259 I 0.837, 842 G 0.518
			f	0.89768	0.06210	0.03761	0.00260		
			ω0	0.03585	1.00000	0.03585	1.00000		
			ω1	0.03585	1.00000	1.00000	1.00000		
Model A null	50	-14752.399709	1					0.998871622	Not Allowed
**Branch b**									
Model A	51	-14754.732863	Site class	0	1	2a	2b		
			f	0.93478	0.06522	0.00000	0.00000		
			ω0	0.03673	1.00000	0.03673	1.00000		
			ω1	0.03673	1.00000	1.00000	1.00000		
Model A null	50	-14754.732884	1					0.994829153	Not Allowed
**Branch c**									
Model A	51	-14754.732897	Site class	0	1	2a	2b		291 K 0.597
			f	0.93478	0.06522	0.00000	0.00000		
			ω0	0.03673	1.00000	0.03673	1.00000		
			ω1	0.03673	1.00000	1.00000	1.00000		
Model A null	50	-14754.732862	1					0.993324497	Not Allowed

Model	np	Ln L	Estimates of parameters					LRT P-value	Positive sites
**Branch d**									
Model A	51	-14754.732868	Site class	0	1	2a	2b		
			f	0.93478	0.06522	0.00000	0.00000		664 K 0.564
			ω0	0.03673	1.00000	0.03673	1.00000		
			ω1	0.03673	1.00000	1.00000	1.00000		
Model A null	50	-14754.732883	1					0.995629828	Not Allowed
**Branch e**									
Model A	51	-14754.732865	Site class	0	1	2a	2b		
			f	0.93479	0.06521	0.00000	0.00000		836 S 0.512
			ω0	0.03673	1.00000	0.03673	1.00000		
			ω1	0.03673	1.00000	1.00000	1.00000		
Model A null	50	-14754.732862	1					0.998045592	Not Allowed
