# Supplementary figures and images for: Molecular adaptations underlying high-frequency hearing in the brain of CF bats species
Source: BMC Genomics. 2024 Mar 16;25:279. doi: 10.1186/s12864-024-10212-6 (PMC10943862; doi:10.1186/s12864-024-10212-6)

Figure S1


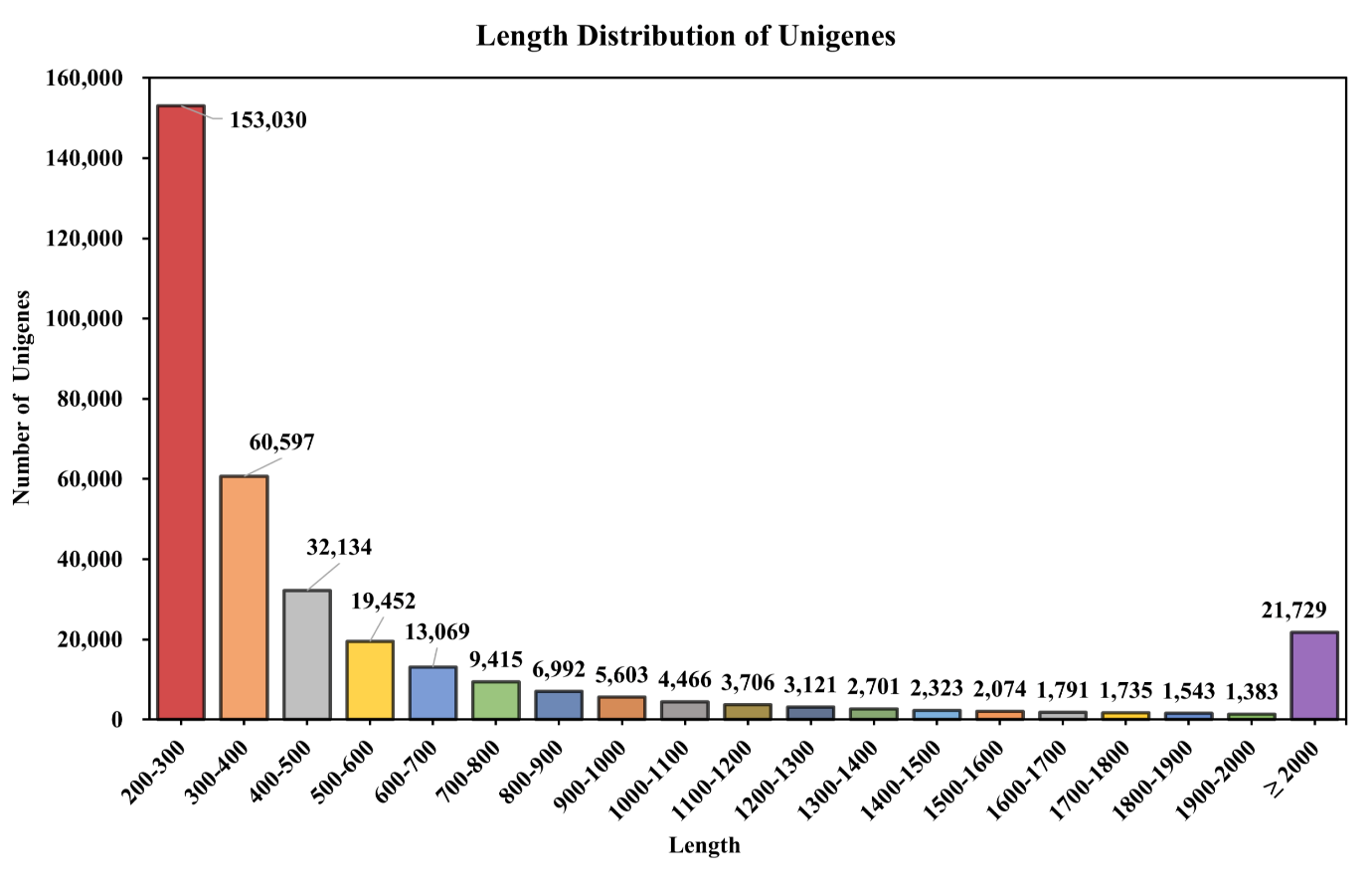


Figure S2


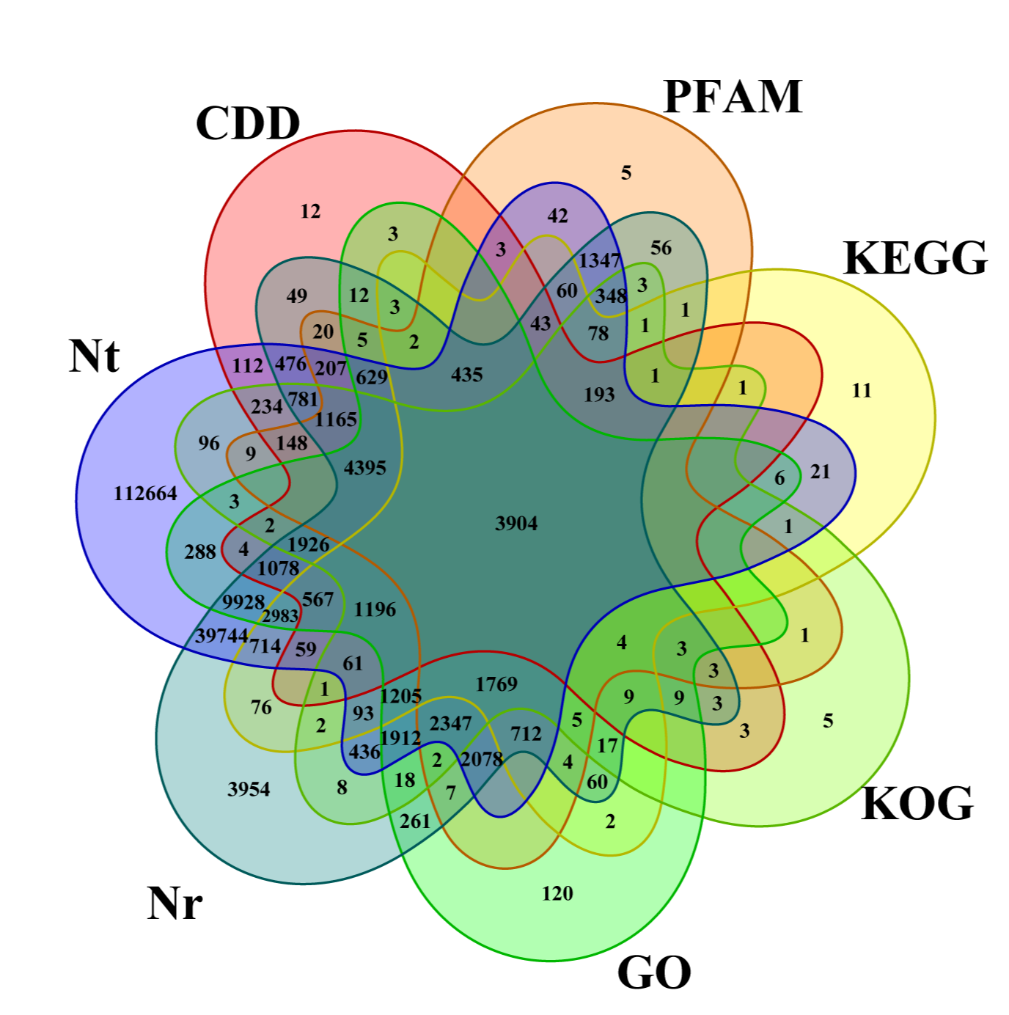


Figure S3


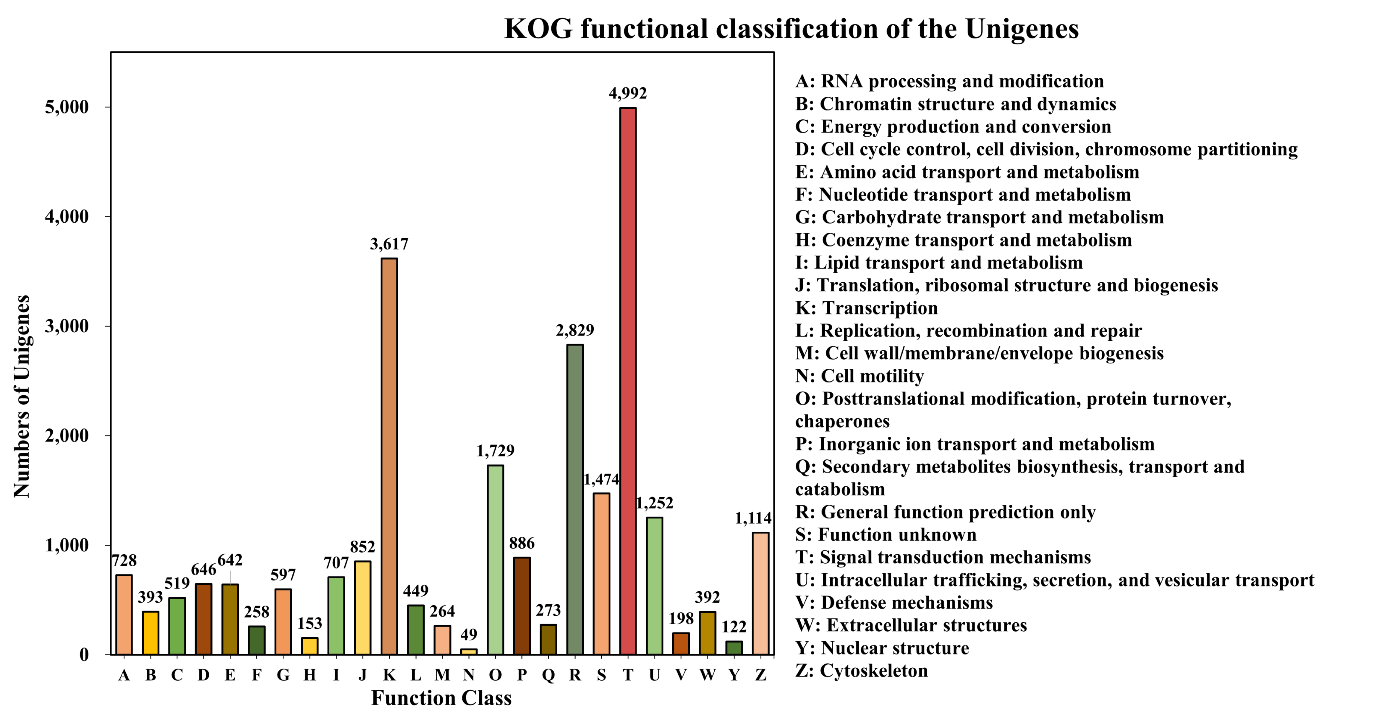


Figure S4


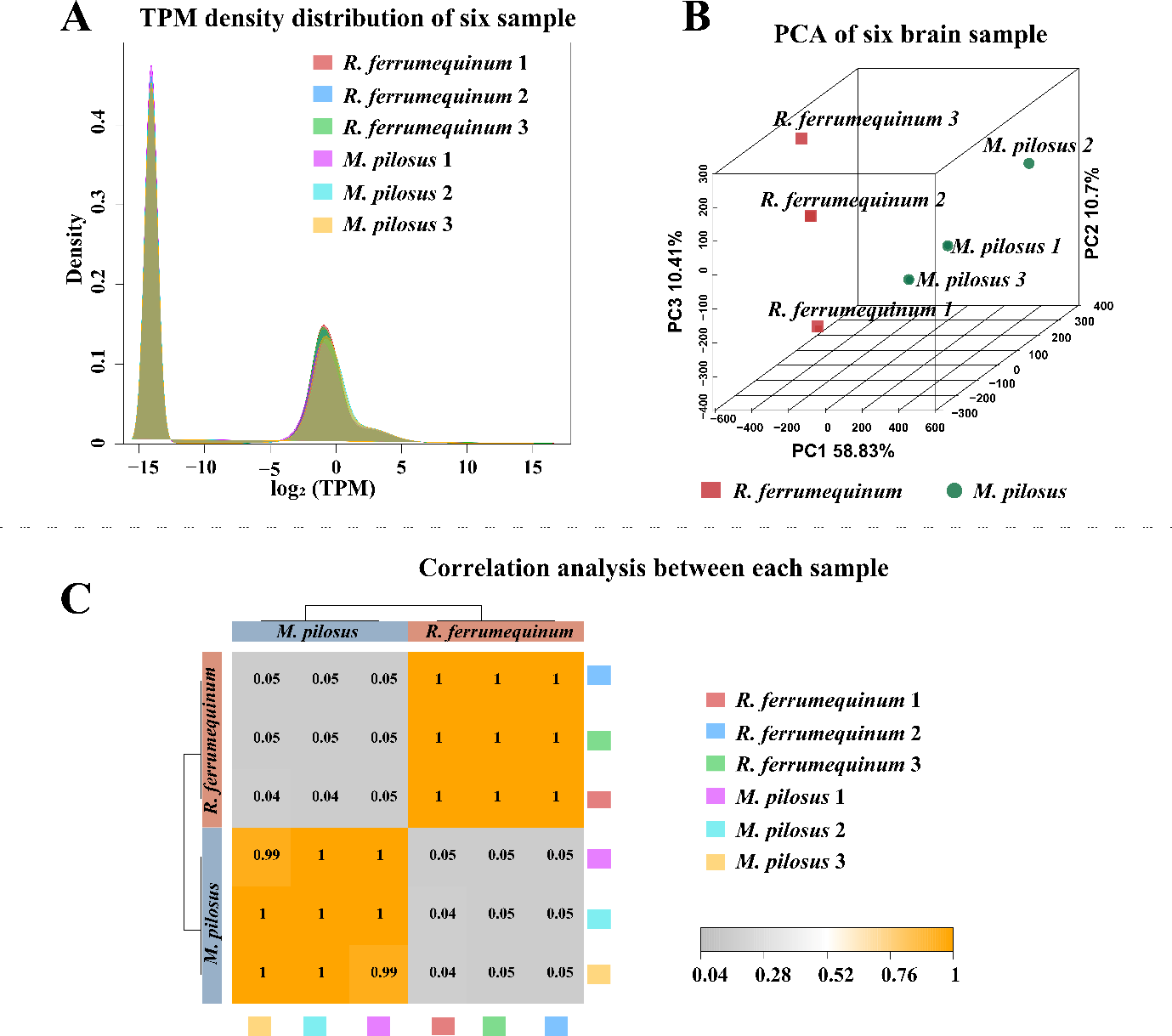


Figure S5


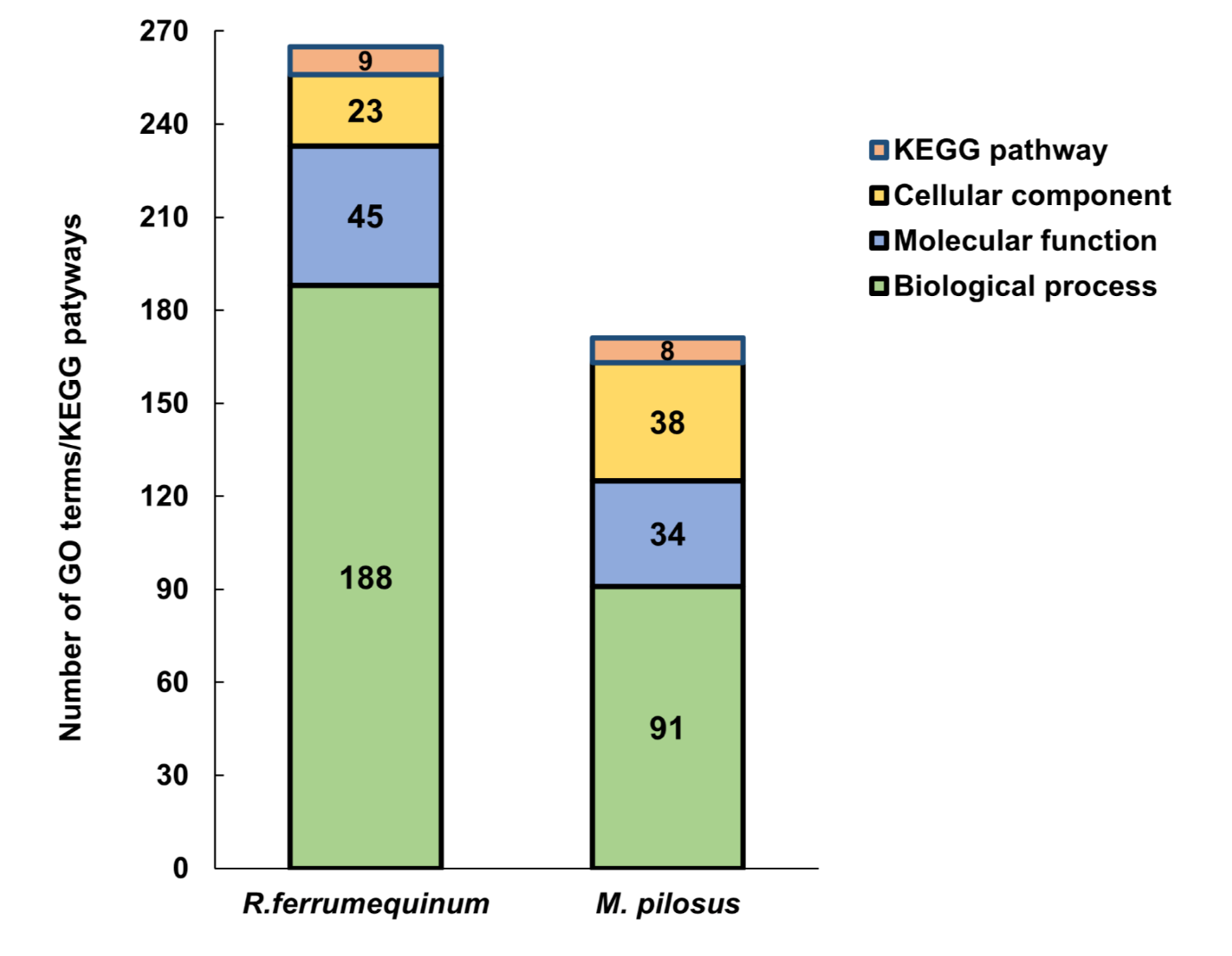

Supplement: Supplementary file 5 — Supplementary Material 5 [file 12864_2024_10212_MOESM5_ESM.docx]
